# Efficacy of upadacitinib in the management of atopic dermatitis, Crohn’s disease, and hidradenitis suppurativa: one treatment, multiple indications^☆^

**DOI:** 10.1016/j.abd.2024.10.007

**Published:** 2025-04-21

**Authors:** Roberto Bueno-Filho, Daniel Lorenzini, Rogério Serafim Parra

**Affiliations:** a Division of Dermatology, Department of Internal Medicine, Faculty of Medicine, Universidade de São Paulo, Ribeirão Preto, SP, Brazil; b Department of Dermatology, Santa Casa de Porto Alegre, Porto Alegre, RS, Brazil; c Division of Coloproctology, Department of Surgery and Anatomy, Faculty of Medicine, Universidade de São Paulo, Ribeirão Preto, SP, Brazil

*Dear Editor,*

Atopic dermatitis (AD) and Crohn's disease (CD) are chronic inflammatory diseases that share pathophysiological similarities, although their immunopathological mechanisms have not been fully elucidated.^1^

Janus kinase inhibitors (JAKi) have proven to be effective in the treatment of inflammatory and autoimmune diseases, including AD and CD.^2^ Moreover, the literature reports a predisposition for a bidirectional association between AD and CD.^1^ This article discusses two cases of the association of AD and CD treated with upadacitinib, achieving control of both diseases.

Case 1 A 47-year-old female patient presented AD and asthma since childhood. At 39 years old, she developed CD, and received mesalazine, prednisone, and antibiotics. In 2019, there was worsening of CD with enterorrhagia, diarrhea, vomiting and weight loss. The fecal calprotectin level was 56 mcg/g (RV < 50 µg/g) and treatment with ustekinumab was started.

In 2021, she had an AD exacerbation, with erythematous-crusted lesions, lichenification and excoriation on the neck, limbs, scalp, and erythematous-desquamative lesions on the hands (Fig. 1A). Disease activity scores were high: Scoring Atopic Dermatitis (SCORAD) of 45 and Dermatology Life Quality Index (DLQI) of 20.

**Fig. 1:**
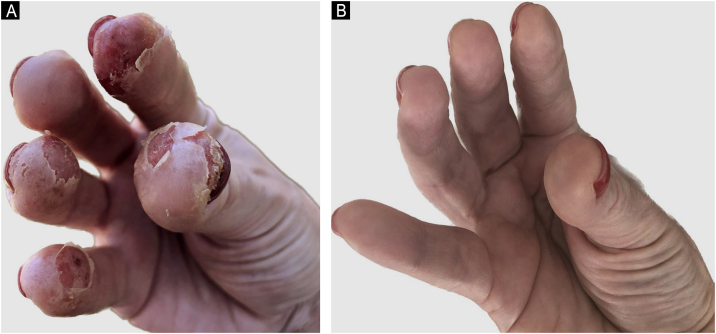
Case 1 (A) shows extensive erythematous and desquamative lesions, especially on the digital pulps of the right hand, with some crusts; (B) complete improvement of the skin lesions after upadacitinib.

She used cyclosporine 300 mg/day with initial improvement but showed a loss of response after three months. Upadacitinib 15 mg/day was initiated with significant improvement in AD and CD, with a fecal calprotectin level of 10 mcg/g. After seven months, ustekinumab was discontinued due to CD control, and she currently has SCORAD of 0 and DLQI of 0 (Fig. 1B).

Case 2 A 19-year-old male patient presented AD and asthma in childhood and was diagnosed with CD in 2015. He had severe acne and hidradenitis suppurativa (HS) in 2019, treated with isotretinoin. He had previously used mesalazine, adalimumab, and prednisone for CD and has been using ustekinumab since 2020.

In 2022, he had simultaneous worsening of AD and HS: fistulized nodules on the face, abdomen, axillae, and buttocks (Fig. 2). He had intense pruritus and exudative erythematous-squamous lesions, on the face, hands, thighs, and feet. The IgE level was 1006 kU/L and fecal calprotectin was >3000 µg/g. DLQI was 18 and SCORAD was 65.4. An ileocolonoscopy showed deep ulcers and inflammation in the right colon (Fig. 3).

**Fig. 2:**
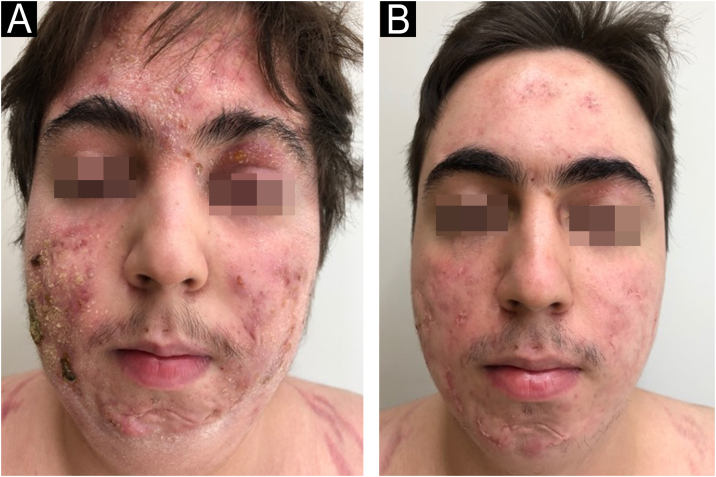
Case 2 (A) multiple lesions with erythema, desquamation, crusts, and also nodules, some containing secretion; (B) almost complete improvement of the inflammation, leaving unaesthetic scars on the face, especially in places where there were nodules.

**Fig. 3:**
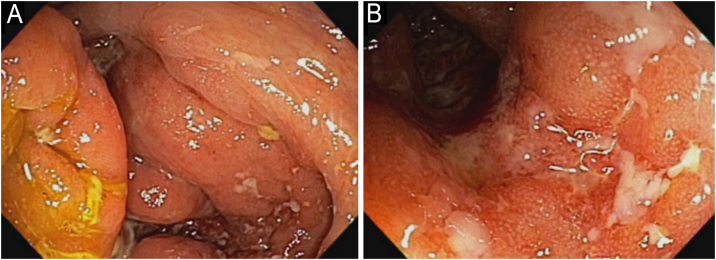
Ileocolonoscopy before upadacitinib treatment. The ileocecal valve (A) and cecum (B) show multiple deep ulcers, indicative of active Crohn’s disease.

Upadacitinib 15 mg/day was started with complete improvement of the skin lesions (Fig. 2). The patient showed clinical remission of CD, with fecal calprotectin normalization at 35 µg/g and ileocolonoscopy showing endoscopic remission (Fig. 4).

**Fig. 4:**
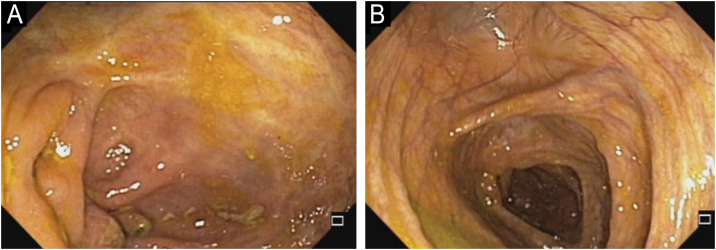
Ileocolonoscopy after treatment with upadacitinib. The ileocecal valve (A) and the cecum (B) show complete mucosal healing.

Upadacitinib is a JAK1i, currently approved for AD treatment in Brazil and for AD and CD in the USA. Despite the absence of protocols/guidelines for the concomitant treatment of multiple inflammatory diseases, the profile of inhibited interleukins potentially encompasses associated diseases.^1^

Mendelian randomization studies and meta-analyses have highlighted a bidirectional relationship between AD and CD and other inflammatory/autoimmune diseases, such as rheumatoid arthritis.^1^ Among the mechanisms that could explain the association are shared predisposing factors such as stress, obesity, lack of breastfeeding, urbanization, and diet.^3^

Inadequate inflammatory responses to intestinal or cutaneous microorganisms, leading to disruption of the external environment protective barrier, be it intestinal epithelium or epidermis, together with dysbiosis and colonization by pathogenic microorganisms, provide possible explanations.^3^, ^4^, ^5^

Genetic predisposition may play a role, as genes that predispose to AD regulate T-cell differentiation and function, as well as certain components of the innate immune system, and some genes are shared with other diseases, such as CD.^6^

The shared T-cell-mediated inflammation in AD and CD is noteworthy: one-third of AD patients exhibit immune autoreactivity, particularly those with chronic or persistent disease.^7^ Exaggerated Th1 and Th17 responses promote autoimmunity and contribute to the chronicity of CD, and are also implicated in AD persistence. It has been speculated that chronic inflammation in AD may foster sustained Th1/Th17 inflammation and predispose to diseases such as CD.^8^, ^9^

The understanding of the pathophysiology and epidemiology does not yet provide a precise explanation for the association between these diseases. However, one must remain vigilant to improve therapeutic approaches. The prospect of treating inflammatory pathologies with a single medication represents remarkable progress, as drug interactions and unwanted side effects are reduced. Although the reported cases demonstrate promising results in the treatment of multiple inflammatory diseases with upadacitinib, further studies are required to confirm its long-term safety and efficacy. Until more evidence emerges, it is essential that therapy be individually and carefully monitored.

## Financial support

None declared.

## Authors’ contributions

Roberto Bueno-Filho: Design and planning of the study; collection of data, or analysis and interpretation of data; drafting and editing of the manuscript or critical review of important intellectual content; collection, analysis and interpretation of data; effective participation in research orientation; intellectual participation in the propaedeutic and/or therapeutic conduct of the studied cases; critical review of the literature; approval of the final version of the manuscript.

Daniel Lorenzini: Design and planning of the study; collection of data, or analysis and interpretation of data; collection, analysis and interpretation of data; intellectual participation in the propaedeutic and/or therapeutic conduct of the studied cases; approval of the final version of the manuscript.

Rogério Serafim Parra: Collection of data, or analysis and interpretation of data; drafting and editing of the manuscript or critical review of important intellectual content; collection, analysis and interpretation of data; intellectual participation in the propaedeutic and/or therapeutic conduct of the studied cases; critical review of the literature; approval of the final version of the manuscript.

## Conflicts of interest

None declared.
